# The New South Wales Mouse Plague 2020-2021: A One Health description

**DOI:** 10.1016/j.onehlt.2024.100753

**Published:** 2024-05-09

**Authors:** Jennifer White, Joanne Taylor, Peter R. Brown, Steve Henry, Lucy Carter, Aditi Mankad, Wei-Shan Chang, Priscilla Stanley, Kerry Collins, David N. Durrheim, Kirrilly Thompson

**Affiliations:** a Health Protection, Hunter New England Local Health District, Booth Building, Wallsend Health Services Longworth Avenue, Wallsend, NSW 2287, Australia; b School of Medicine and Public Health, University of Newcastle, Callaghan, NSW 2308, Australia; c CSIRO Health and Biosecurity, GPO Box 1700, Canberra, ACT 2601, Australia; d CSIRO Environment, GPO Box 2583, Brisbane, QLD 4001, Australia; e Western NSW Local Health District, PO Box 4061, Dubbo, NSW 2830, Australia; f Flinders University, Sturt Rd, Bedford Park, SA 5042, Australia

**Keywords:** Australia, Disaster, Encephalomyocarditis virus, Leptospirosis, Lymphocytic choriomeningitis virus, Mental health, Mouse plague, One health, Poisoning, Rodenticide, Stress

## Abstract

A mouse plague occurred in Eastern Australia from spring 2020 to winter 2021, impacting an area of around 180,000 km^2^. It harmed human physical and psychological health, damaged the natural and built environment, and endangered farmed, domestic and native animals. However, the mouse plague was overshadowed by the COVID-19 pandemic, especially as the end of the plague coincided with the arrival and surge of the COVID-19 delta strain in rural New South Wales (NSW). In this article, we systematically overview the multiple impacts of the plague and highlight their complex interactions. Using a One Health framework, we comprehensively review the i) human, ii) animal and iii) environmental impacts including economic dimensions. Given the damage that the mouse plague caused to infrastructure, we consider the environment from two perspectives: the natural and the built environment. This One Health description of the 2020–2021 mouse plague identifies priorities for preparedness, response and recovery at local, regional land levels to inform response and management of future mouse plague events in Australia. It also highlights the need for ongoing collaboration between researchers and practitioners in the human, animal and environmental health sectors.

## Highlights

• A large-scale mouse plague impacted Eastern Australia during the COVID-19 pandemic.
• This is the first study to investigate mouse plagues using a One health approach.
• This enabled the identification of interrelations between profound human, animal, environmental and economic impacts.
• A One Health approach is proposed for managing and responding to future mouse plague events.

## 1 Introduction

Mouse plagues usually occur in association with a constellation of environmental conditions that result in population increases from 5 mice/ha (ha) to well over 1000 mice/ha in a 12–18 month period [1]. Mouse plagues have been a feature of Australian cereal cropping regions since the 1800s with the worst recorded plague to date occurring across South Australia (SA) and Victoria (VIC) in 1993. Across Australia, records show that mouse plagues occur every four or five years [2] and even as frequently as every three years in Queensland (QLD) [3]. Predisposing environmental conditions include longer term weather patterns, especially above average rainfall, increased food supply, reduced predation and mouse diseases, and changes in the social structure of mice [4]. Heavy rainfall, in particular, has been identified as a key predisposing feature of mouse plagues, especially in the preceding 1–2 years and after a series of dry years [1].

**Fig. 1:**
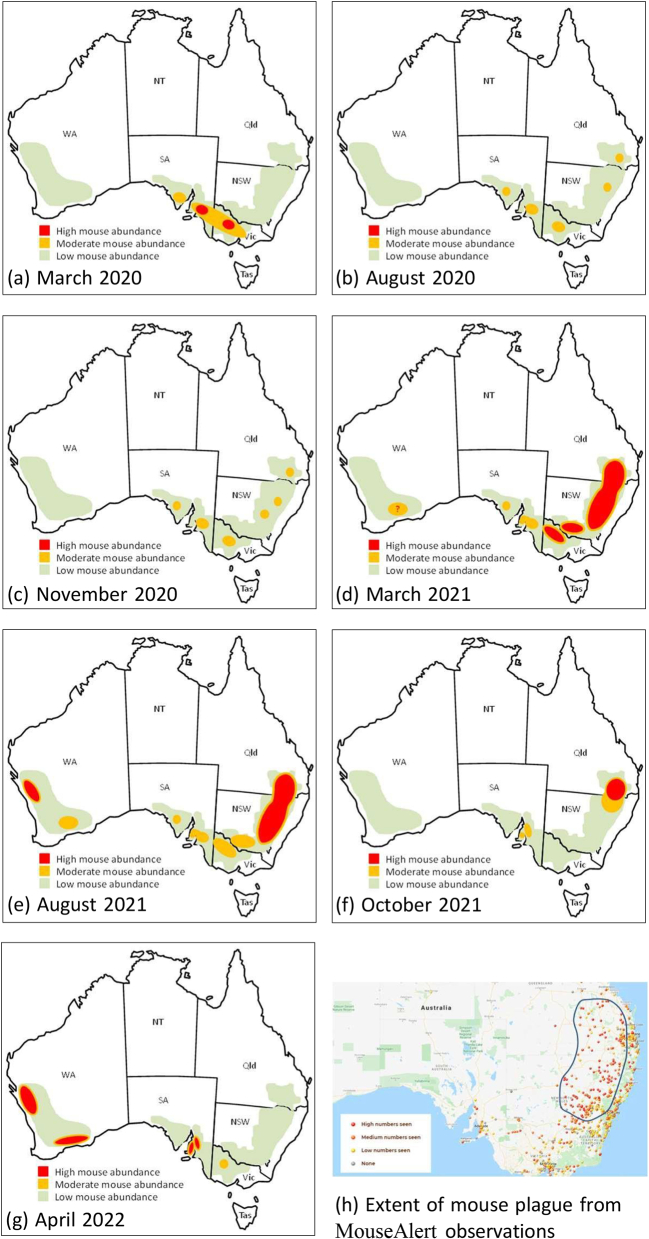
Australia (New South Wales and southern Queensland) from prior to the plague (March 2020) through to after the plague had ended (April 2022). These maps were drawn from the *Mouse Updates* produced by CSIRO (issued three times a year) from the Grains Research and Development Corporation (GRDC) project to monitor mouse populations across Australia. It incorporates data from a range of sources (trapping data from 4 benchmark sites, rapid assessment data using mouse chew cards and active burrow counts from ∼130 sites, and reports from farmers, agronomists and other networks). All reports are available at: https://research.csiro.au/rm/mouse-activity-updates/. Also shown is data from MouseAlert (https://www.feralscan.org.au/mousealert/) which was used to define the area where the mouse plague was most severe (central and northern NSW and south-eastern Queensland) in 2021. Australia (New South Wales and southern Queensland) from prior to the plague (March 2020) through to after the plague had ended (April 2022). These maps were drawn from the *Mouse Updates* produced by CSIRO (issued three times a year) from the Grains Research and Development Corporation (GRDC) project to monitor mouse populations across Australia. It incorporates data from a range of sources (trapping data from 4 benchmark sites, rapid assessment data using mouse chew cards and active burrow counts from ∼130 sites, and reports from farmers, agronomists and other networks). All reports are available at: https://research.csiro.au/rm/mouse-activity-updates/. Also shown is data from MouseAlert (https://www.feralscan.org.au/mousealert/) which was used to define the area where the mouse plague was most severe (central and northern NSW and south-eastern Queensland) in 2021.

A system for monitoring mouse populations in Australia has been in place since 2012, and has grown to approximately 150 sites across 15 regions in five Australian states [5]. Monitoring is funded by the Grains Research and Development Corporation (GRDC) and is coordinated by the Commonwealth Scientific and Industrial Research Organisation (CSIRO) [6]. This monitoring proved integral in tracking the evolution of the 2020 mouse plague in New South Wales (NSW). During 2020 and 2021, mouse numbers burgeoned across large parts of central and northern NSW following exceptional rainfall after several years of drought. While moderate levels of mouse activity were detected in parts of northern NSW from August 2020 and again in November 2020 (Fig. 1(b) and 1(c)), by March 2021, numbers were exceptionally high across a broad area from central NSW extending up through to southern QLD on the Darling Downs (Fig. 1(d)). Mouse numbers remained high in nearly all areas until August 2021 (Fig. 1(e)). They then precipitously fell by October in most areas in central and northern NSW (Fig. 1(f)), with high numbers persisting for a little longer in southern QLD.

The reasons for the dramatic decrease in mouse numbers from October 2021 are not well understood, but disease, increased social pressure and reduced food supply are considered important factors underlying the end of mouse plagues [7].

Mouse plagues impact on and are impacted by humans, animals and their shared environment. As a result, they are suitably contextualised within a One Health philosophy which recognises how, “the health of humans, domestic and wild animals, plants, and the wider environment (including ecosystems) are closely linked and interdependent’ [8]. In this paper, we adopt a One Health framework to describe the 2020/2021 NSW mouse plague and include a discussion of economic impacts, to account for the significant monetary and human emotional costs. Our separation of humans, animals and the environment is largely academic but facilitates information presentation. In reality, they are interdependent, as recognised by a One Health approach.

## 2 Mouse plague impacts

### 2.1 Human

#### 2.1.1 Physical health impacts

Several physical impacts to people were prominent during the mouse plague. The first relates to rodenticide exposure. Anticoagulant rodenticides are registered by the Australian Pesticides and Veterinary Medicines Authority (APVMA), but can only be used in and around buildings or domestic use (not indoors), and/or in bait stations, or by professional pest control operators (for details see https://portal.apvma.gov.au/pubcris). Zinc phosphide bait is the only rodenticide that is registered for use in broadacre (cropping) systems in Australia [[9], [10], [11]]. During the 2020/2021 plague, the APVMA granted an emergency use permit (PER90799, 7 May 2021 to 31 December 2023) and rebate scheme (via the NSW Government) to increase the concentration of ZnP from 25 g ZnP/kg to 50 g ZnP/kg in grain bait products. However, suspected poisoning occurred in seven individuals who either presented to a health facility or called the Poisons Information Centre with symptoms consistent with poisoning from zinc phosphide bait (ZnP). Three cases disclosed the use of zinc phosphide indoors. Five individuals were hospitalised and all recovered.

**Fig. 2:**
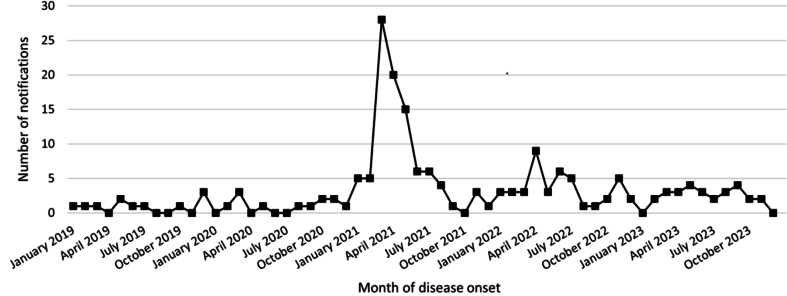
Number of cases of Leptospirosis each month in NSW from 2019 to 2023 showing an increase in number of infections coincident with the mouse plague in 2021.

The second human health impact of mouse plague related to the zoonotic pathogens carried by mice. There were 94 officially notified cases of *Leptospira spp* (leptospirosis) across NSW in 2021, compared to an average of 21 cases per annum over the previous decade (see Fig. 2). *Lymphocytic choriomeningitis virus* (LCMV), a rare and potentially life-threatening pathogen, was identified in one 51 year-old farmer in Western NSW [12] and a cluster of 8 cases (age range 4–72 years) were identified in South-East Queensland during the first 6 months of 2021 [13]. These were the first ever noted cases of LCMV in these areas.

#### 2.1.2 Psychological and mental health impacts

During the 2020/2021 mouse plague, a media report compared the mental health impact with that of a natural disaster [14]. According to the American Psychological Association, chronic stress associated with a natural disaster is characterised by constant and persistent symptoms such as anxiety, insomnia and high blood pressure [15]. There remains limited empirical published data concerning the mental health and psychological impacts of mouse plagues in the Australian context. Internationally, research on the broader social impacts of mice and rodent infestation has been linked to social disadvantage, including poor housing quality, and an increased likelihood of experiencing symptoms of mental illness [16].

During the 2020/2021 mouse plague, a NSW Farmers Association survey [17] (in conjunction with the Country Women's Association) found that 97% of respondents reported increased stress levels and difficulty sleeping. Northern NSW media reports noted the experience of constant fatigue, frustration and repulsion at managing the plague, alongside sleep disturbance and hyper vigilance [14]. These findings are consistent with a study which explored the impact of constant exposure to rats and found poor sleep quality with some people adopting warning systems (e.g. traps) to alert them of nearby rats, and modifying their sleeping behaviours (e.g., wrapping selves in blankets with only a vent hole for breathing) [18]. Rodent exposure has been associated with feelings of depression [19], physiological symptoms (headaches, dizziness and nausea [18]) and visceral reactions to the potential or actual presence of mice - being considered dirty, a source of disease and a threat to safety [20]. Frequent exposure to rodents can lead to *musophobia*, the extreme fear of mice and rats [21]. Evidence indicates that these kinds of negative emotions endure long term [22].

While the cognitive or emotional effects of killing mice during a plague have not been empirically explored, the psychological burden of killing mice has been examined in the context of laboratory workers who killed mice as part of their vocation [23]. In that study, laboratory researchers were reported to be emotionally and physically overwhelmed, and “morally exhausted” [23]. Although not a direct comparison, the literature suggests that the repeated killing of mice can have a negative impact on one's wellbeing. The effects may also be heightened if the burden of killing mice is combined with other mental loads associated with managing a plague in the home, such as cleaning, disease prevention and loss of income. Given that mouse plagues often follow drought conditions, the cumulative effects of stress can have catastrophic impacts on mental health.

### 2.2 Animals

#### 2.2.1 Livestock

Mouse plagues present significant potential impacts to the livestock industry as feed and water sources can become contaminated with mouse urine and increase the risk of *leptospirosis* in sheep and cattle [24]. Depending on the strain of the disease, *leptospirosis* can cause abortions and reproductive losses in cattle. Livestock are also at risk of botulism associated with consumption of mouse carcases in fodder [24].

In 2021, there were outbreaks of *encephalomyocarditis virus* (EMCV) infection in pigs. EMCV can have severe consequences and result in death or reproductive problems. There is currently no treatment for EMCV. In early 2021, EMCV caused the death of 68 piglets (aged 6 weeks) in the Upper Hunter Valley [25] and 24 piglets (aged 6 weeks) in another Hunter region [26]. This result demonstrates the eastward expansion of the plague towards the Pacific Coast. Previously mouse plagues have largely impacted west of the Great Dividing Range in NSW, but the Upper Hunter Valley is east of the Range. By mid-2021, EMCV was confirmed in two separate piggeries in NSW [25]. Deaths ceased after the contaminated feed was removed and extensive rodent control was implemented.

Between December 2020 and February 2021 *Yersinia enterocolitica* was diagnosed in a herd of 260 goats in north-western NSW, coinciding with the mouse plague [25]. Fourteen goats were affected and eight goats died [25].

*Leptospirosis*, ECMV and yersiniosis are zoonotic diseases; the increase of these diseases in livestock is concerning as people who come into contact with infected animals can be infected [27]. As the bacterium enters the body both through skin cuts or abrasions from contaminated soil or mud, or through ingesting contaminated water, broad environmental contamination increases the risk of infection [27].

#### 2.2.2 Pets and wildlife

There is some suggestion in the literature that mice plagues lead to an increase in a range of species such as birds of prey as well as snakes, feral cats and foxes and this increased food availability [28].

Exposure to rodenticide can also result in pet and wildlife death due to primary or secondary toxicity. Primary toxicity occurs when pets consume baited material, including zinc phosphide baited grain or anti-coagulant rodenticide baits [29]. Secondary toxicity occurs from the accumulation of ingested anti-coagulant chemicals through the food chain [29]. There can also be impacts on native wildlife from rodenticide toxicity or when farmers go to extreme lengths to control mice such as using arsenic [30]. It is difficult to confirm poisoning events in native animals as not all wildlife poisoning events are reported [31]. However, during the first six months of 2021, reported cases of rodenticide toxicity in birds increased and exceeded the total number of cases reported by the NSW Environment Protection Authority (EPA) in 2020 [29].

A survey (designed by Birdlife Australia and circulated by the Australian Veterinary Association) conducted between September 2021 and January 2022 found more than nine in ten Australian vets had treated pets for poisoning from anti-coagulant rodenticide products commonly supplied by supermarkets [32].

Most research on the link between human and animal wellbeing is concerned with the benefits of animals to human wellbeing, rather than the impact of compromised human wellbeing on that of animal wellbeing. Whilst we did not locate any specific reports of altered animal behaviour indicative of compromised wellbeing, it is reasonable to expect that the wellbeing of pets and wildlife was impacted by the mouse plague. Certainly, the cognitive, emotional and practical burden of experiencing and responding to the mouse plague may have compromised the ability of animal owners and carers to maintain their usual levels of care and attention to pets, livestock and any wildlife to which they may have otherwise offered assistance. Humans and animals are sensitive to changes in the environment as well as changes to ‘usual’ patterns of activity, which can impact their neuroception of safety. In particular, domestic animals may respond to the increased levels of stress, anxiety and dysregulation they perceive in the humans with which they interact. These stress-related states amongst humans and/or their animals may have impacted any interspecies co-regulation they might have usually experienced [33].

### 2.3 Environment

#### 2.3.1 Natural environment and farming

The 2020/2021 mouse plague had a devastating impact on the natural environment, with attendant economic impacts. Specifically, the mouse plague impeded production of a variety of cereal, pulse and horticultural crops in Australia (e.g., wheat, sorghum, lupins, canola, soybeans, cotton, melons) [7]. Consistent with past research, crop damage due to the mouse plague occurred at all stages of growth, from feeding on seed or newly emerging seedlings impacting crop germination, to eating unharvested grain with reduced yield, and chewing growing nodes of plants thus affecting plant development and structure [3,7]. In addition, there were extensive reports of loss, damage or contamination of stored grain and fodder, and damage to the strings and the structure of bales making them difficult to transport and sell [7,17].

Mouse plague events have previously been averaged to cost approximately A$20 million a year [34] and to occur every four to five years [2]. However, a careful assessment of the direct economic impact of the 1993 mouse plague that affected South Australia and Victoria conservatively estimated A$64.5 million in direct losses [35]. The 2020/21 mouse plague experience indicates that the costs are likely to be much greater, with the NSW Farmers Federation estimating the cost at A$1 billion due to damage to agricultural crops including consumption of newly planted seed and maturing crops, consumption and fouling of stored grain and stockfeed, and damage to farm equipment, buildings, and farmsteads [36,37]. Additional costs varied between growers and were estimated at A$140,000 per grower on average (range = A$7000–461,580; equivalent to 30–40% loss of value) due to damage of stored fodder, cost of rodenticides, and labour costs [38]. The NSW Farmers Association survey identified that 30% of respondents spent between A$50,000–150,000 on baiting, 80% of respondents spent between A$20,000–150,000 in addressing damage to agricultural machinery and infrastructure, and approximately one third reported that their greatest cost (between $50,000 and $150,000) was due to damaged grain and fodder. Further, many farmers used crop duster planes to lay bait, with attendant significant cost [17,39].

Closely associated with the natural environment were the impacts on domestic water storage systems and rainwater tanks that were either damaged by or contaminated with excretions from live mice or the bodies of dead mice [40]. Anecdotal reports suggested that damage was extensive and that local residents found it impossible to keep mice out of their water tanks, not just contamination from urine and faeces but dead and drowning mice. Indeed, the pervasive smell of mice is a known source of psychological distress [20].

#### 2.3.2 Built environment and communities

Multiple media reports identified the diverse impacts of the mouse plague on the broader community resulting from damage to the built environment including council property, schools, libraries, childcare centres and aged care homes [[41], [42], [43], [44], [45]]; local swimming and sporting centres, sewer works; and supply losses in supermarket/retail, hotel, retail and food businesses [46,47]. Likewise, there were widespread reports of damage to vehicles and home structures [38]. Local hospitals experienced unprecedented occupational health and safety challenges including increased baiting and trapping, use of odour repellents, increased frequency of food waste removal, improving seals around doors and windows, yard and grass clearing around buildings, blocking of brickwork weepholes and other cavities [48]. The damage to building infrastructure required inmates from a large Correctional Service Centre to be relocated until repairs could be completed [[49], [50], [51]]. It is possible that residents may have been displaced if unable to seal their home adequately.

An additional financial toll was attributed to the lack of home insurance policy coverage for plague-related damage except when resulting from fires [52].

### 2.4 Regional response

Despite facing the demands of an emerging global pandemic, there was wide-scale communication of mouse management approaches provided through trusted rural media sources and on-line [53]. Government subsidy of preferred rodenticides and warnings on their safe use were also broadcast. Local community events were run through trusted local landcare organisations and were facilitated by experts in mouse control, veterinary and human health. The Salvation Army, a charitable organisation, with a particular focus on mental health crisis support, was also present at these events.

Health advice was provided to the broader community through broadcast and print media and on-line factsheets with a particular focus on those infectious diseases resulting from direct contact with mice or the environment/food contaminated with mouse urine or faeces especially *leptospirosis*, rat bite fever, LCM and *salmonellosis*. Guidance on avoiding mouse contact (where possible), appropriate management of bites, and ensuring the safety of food and water (including water tanks) was also provided.

There was heightened awareness and surveillance in hospital emergency departments through free-text triage for mouse-related infections, with clinical alerts to hospital clinicians, including those in emergency, neurology and infectious diseases departments, and to primary care providers.

## 3 Discussion

**Fig. 3:**
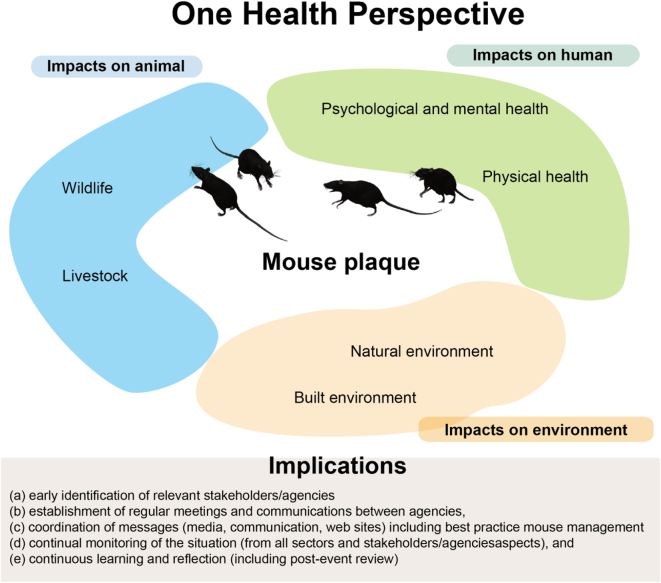
One Health Perspective. Summary of mouse plaque using One Health perspective, including impacts on human, animal, environment and future implications.

The significant impact of the mouse plague highlighted in this paper makes this a critical area for ongoing multidisciplinary collaborations that combine preventive measures, monitoring, effective communication, and coordinated control strategies. While it is important to invest in research regarding the ecology and behaviour of mice, a One Health approach should also be the basis for early detection and monitoring and coordinated prevention and control. Furthermore, we advocate that a One Health approach is ideally positioned to facilitate participatory and collaborative work that promotes education and community preparedness for future plagues. Based on key findings from this paper, a One Health approach would entail: (a) early identification of relevant partners/agencies (e.g. government departments, researchers, non-government organisations, veterinarians, general practitioners, emergency departments, wildlife carers, farmer groups), (b) establishment of regular meetings and communications between agencies, (c) coordination of messages (media, communication, websites) including best practice mouse management to reduce mouse damage in fields, but also in homes and sheds – whilst mitigating the risk of poisoning to humans or animals, (d) continual monitoring of the situation (by all sectors and stakeholders/agencies), and (e) continuous learning and reflection (including post-event review). (Fig. 3).

In response to the mouse plague, methods for improving monitoring (including real-time monitoring) and improved forecast models are currently underway (funded by the GRDC). Future research is needed to explore the impact of the mouse plague on community cohesion, resilience, and wellbeing as these may impact local responses to future events in the region.

This description of the 2020/2021 mouse plague in eastern Australia clearly highlights the multiple and complex intersections between human, animal and environmental domains that can be captured by a One Health approach.

## Consent for publication

Not applicable.

## CRediT authorship contribution statement

**Jennifer White:** Writing – original draft, Writing – review & editing, Investigation, Methodology, Project administration. **Joanne Taylor:** Conceptualization, Writing – original draft, Writing – review & editing, Investigation, Methodology, Project administration, Supervision. **Peter R. Brown:** Writing – review & editing, Investigation, Methodology, Writing – original draft. **Steve Henry:** Writing – review & editing, Investigation, Methodology, Writing – original draft. **Lucy Carter:** Writing – review & editing, Investigation, Methodology, Writing – original draft. **Aditi Mankad:** Writing – review & editing, Investigation, Methodology, Writing – original draft. **Wei-Shan Chang:** Writing – review & editing, Investigation, Methodology, Writing – original draft. **Priscilla Stanley:** Writing – review & editing, Investigation, Methodology, Writing – original draft. **Kerry Collins:** Writing – review & editing, Investigation, Methodology, Writing – original draft. **David N. Durrheim:** Conceptualization, Writing – original draft, Writing – review & editing, Investigation, Methodology, Project administration, Supervision. **Kirrilly Thompson:** Conceptualization, Writing – original draft, Writing – review & editing, Investigation, Methodology, Project administration, Supervision.

## Funding

The study was conducted with funding from Hunter New England Local Health District, and JW was funded by New South Wales Health through the Prevention Research Support Fellowship. We acknowledge the Department of Regional NSW and CSIRO for partial financial assistance.

## Declaration of competing interest

The authors declare there is no conflict of interest.

## Data Availability

Data will be made available on request.
